# Modeling the Spread of COVID-19 Infection Using a Multilayer Perceptron

**DOI:** 10.1155/2020/5714714

**Published:** 2020-05-29

**Authors:** Zlatan Car, Sandi Baressi Šegota, Nikola Anđelić, Ivan Lorencin, Vedran Mrzljak

**Affiliations:** Faculty of Engineering Rijeka, University of Rijeka, Vukovarska 58, 51000 Rijeka, Croatia

## Abstract

Coronavirus (COVID-19) is a highly infectious disease that has captured the attention of the worldwide public. Modeling of such diseases can be extremely important in the prediction of their impact. While classic, statistical, modeling can provide satisfactory models, it can also fail to comprehend the intricacies contained within the data. In this paper, authors use a publicly available dataset, containing information on infected, recovered, and deceased patients in 406 locations over 51 days (22nd January 2020 to 12th March 2020). This dataset, intended to be a time-series dataset, is transformed into a regression dataset and used in training a multilayer perceptron (MLP) artificial neural network (ANN). The aim of training is to achieve a worldwide model of the maximal number of patients across all locations in each time unit. Hyperparameters of the MLP are varied using a grid search algorithm, with a total of 5376 hyperparameter combinations. Using those combinations, a total of 48384 ANNs are trained (16128 for each patient group—deceased, recovered, and infected), and each model is evaluated using the coefficient of determination (*R*2). Cross-validation is performed using K-fold algorithm with 5-folds. Best models achieved consists of 4 hidden layers with 4 neurons in each of those layers, and use a ReLU activation function, with *R*2 scores of 0.98599 for confirmed, 0.99429 for deceased, and 0.97941 for recovered patient models. When cross-validation is performed, these scores drop to 0.94 for confirmed, 0.781 for recovered, and 0.986 for deceased patient models, showing high robustness of the deceased patient model, good robustness for confirmed, and low robustness for recovered patient model.

## 1. Introduction

Coronavirus disease, code-named COVID-19, is an infectious disease caused by a virus, a member of the Betacoronavirus family named severe acute respiratory syndrome coronavirus 2 (SARS-CoV-2), previously referred to as 2019 novel coronavirus (2019-nCoV) [1, 2]. It is thought that the virus outbreak has animal origins, and it was first transmitted to humans in Wuhan province China, in November/December 2019 [3–5].

At present, no approved vaccines or specific antivirals are available for COVID-19 [6, 7]. Previous SARS pandemic in 2002 and 2003 was controlled and finally stopped by conventional control measures, including travel restrictions and patient isolation. Currently, these measures are applied in almost all countries with the COVID-19 outbreak; however, their effectiveness depends on how rigorous they are [8, 9]. It follows that the methods enabling reliable prediction of spreading of COVID-19 would be of great benefit in persuading public opinion why it is crucial to adhere to these measures in the past decade [10, 11].

Modeling viral diseases such as COVID-19 is extremely important in determining their possible future impact. Modeling the spread and the effect of such a disease can be supremely important in understanding its impact [12]. While traditional, statistical, modeling can offer precise models [13], artificial intelligence (AI) techniques could be the key to finding high-quality predictive models [14]. In this paper, the authors present a machine learning solution, a multilayer perceptron (MLP) artificial neural network (ANN) [15], to model the spread of the disease, which predicts the maximal number of people who contracted the disease per location in each time unit, maximal number of people who recovered per location in each time unit, and maximal number of deaths per location in each time unit. MLP has been selected for its simplicity in comparison to other AI algorithms, due to authors wishing to test the possibility of modeling using comparatively simple methods, due to shorter training time associated with such methods, because the quick generation of results is important when modeling diseases, due to the as-fast-as-possible requirement for models with good enough regression performance. Modeling can be done on existing data, using statistical analyses. But, when it comes to extremely complex models, statistical analysis can fail to comprehend the intricacies contained in the analyzed data [16]. More complex algorithms, namely, AI algorithms and especially machine learning algorithms can be used to “learn” not just the general trend, but the intricacies of the data, which results in higher quality models produced [10]. AI algorithms have become increasingly applicable in various branches of science and industry, i.e., medicine [17] for the classification of various diseases as well as creating regression models for estimation and prediction. Models obtained by machine learning techniques adjust their parameters to fit their predictions to existing data, no matter what it contains. By doing this, the models take into account interinfluences of various input parameters that might not have been taken into consideration if traditional modeling methods were used [11]. This ability to take into account hard to observe intricacies stored inside data should lend itself well, when used in an attempt of regressing a complex model such as spread of COVID-19. Currently, existing models of COVID-19 spread have relatively poor results [18] or have made predictions which were proven to not correlate to real data [19, 20].

In the research presented, the aim was to achieve an accurate regression model through the utilization of an AI algorithm using the data that existed during the time in which this research was performed. This was done in order to demonstrate the possibility of using AI algorithms in early modeling of infective disease, such as COVID-19, spread. The aim of the model is to observe all the collected data together, instead of separating it into localities, as that mode of observation could allow a machine learning method to achieve a better global model of viral spread. MLP algorithm is trained using a “Novel Coronavirus (COVID-19) Cases” [21], by John Hopkins CSSE. At the time of this research being performed, the dataset contained 20706 data points and was split into the training (75%—15530 data points) and testing (25%—5176 data points) sets. The hyperparameters of the MLP are determined using a grid search algorithm. The robustness of the different models is tested using K-fold cross-validation algorithm. Achieved results are then evaluated using the *R*2 metric. A detailed look upon the techniques used has been given in Materials and Methods.

## 2. Materials and Methods

**Figure 1:**
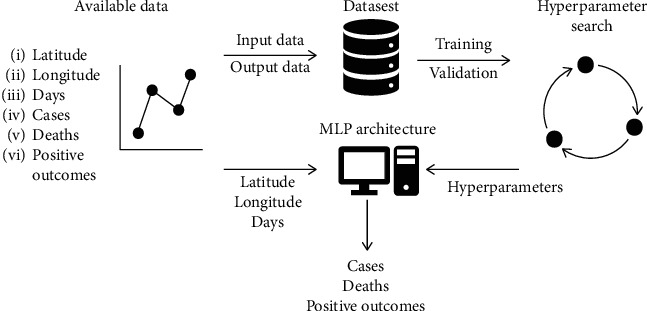
The process of AI modeling is shown. First, the data is collected and placed into a dataset. Part of that data is used for training and testing the various MLP hyperparameter combinations, in an attempt to find the best possible architecture. The most successful model can then be used to determine the future instances.

Materials and methods used in the research are presented in this section. The process from using and transforming the available data, modeling it using MLP with a multitude of hyperparameter combinations, and the final evaluation of results is described. The overview of the modeling process is given in Figure 1.

### 2.1. Dataset Description

**Figure 2:**
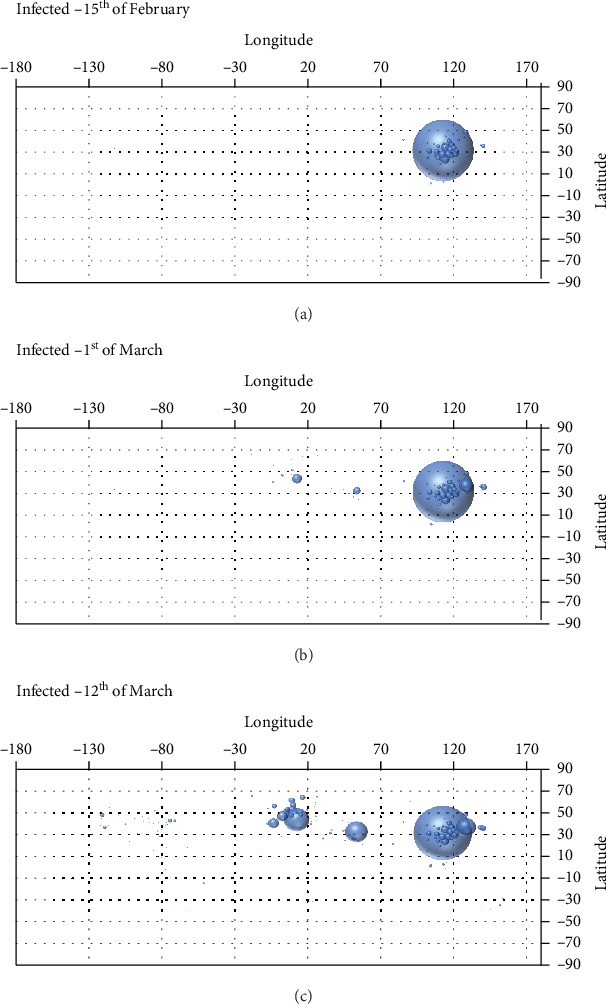
Overview of geographical distributions for number of patients infected with COVID-19 at 15^th^ of February 2020 (a), 1st of March 2020 (b), and 12^th^ of March 2020 (c).

Dataset used in this research is obtained from a publicly available repository operated by the Johns Hopkins University Center for Systems Science and Engineering (JHU CSSE) and supported by ESRI Living Atlas Team and the Johns Hopkins University Applied Physics Lab (JHU APL) [21]. It contains the data for the coronavirus patients which describe the number of patients in a certain location (defined by the name of location, latitude, and longitude), for each day since the start of the COVID-19 infections (22^nd^ of January 2020) until 12^th^ of March 2020. Dataset is split into three groups—infected, recovered, and deceased. At the time of this research being performed, the dataset contained the data for 406 locations and 51 days. The geographical distribution of data contained in the dataset is given in Figure 2, which shows the geographical distribution of infected patients at various points in time.

Dataset, as published, is organized as time-series data—showing the spread of disease in various locations over time. The data collected at the time of this research being performed was insufficient to attempt a time-series AI modeling. To train the MLP, the dataset is rearranged to create a set of inputs and outputs. For each number of cases, the latitude and longitude of the location, as well as the date of data collection is added. The date is converted into the number of days since the first entry into the dataset. In this way, each data point contains information about the number of patients (contracted, recovered, or dead) at a given location, at a given day since the first noted case. Latitude, longitude, and the number of days since the first case are used as input data, with the output data being the number of patients in each group. In this manner, the time-series dataset is rearranged in a manner that makes it appropriate to train a regressive MLP.

Finally, the dataset, consisting of a total of 20706 data points, is randomly split into five equal parts, or so-called folds. Each of these parts is used as a testing set, with the remaining parts used as a training set. This means that training for each architecture is repeated 5 times, with an 80%/20% (16565 randomly selected data points for training and 4141 data points for testing set) training-testing distribution.

### 2.2. Multilayer Perceptron

Multilayer perceptron (MLP) is a type of a fully connected, feed-forward artificial neural network (ANN), consisting of neurons arranged in layers [11]. At least three layers make up MLP: an input layer, an output layer, and one or more hidden layers. The output layer consists of a single neuron, the value of which is the output of the MLP ANN—in the presented research this is the predicted number of patients. The input layer consists of the neurons in the same number as the dataset inputs [22]. MLPs used in this research will as such have 3 neurons in the input layer—one for each of the input data points (latitude, longitude, days since infection).

The reason for selecting MLP as the method used in this research was the ease of implementation of such methods. MLP is also known to provide high-quality models, while keeping the training time relatively low compared to more complex methods.

MLP is based on calculating the values of neurons in a current layer as the activated summation of weighted outputs of neurons in a previous layer, connected to the neuron [22, 23]. Activation refers to the sums of weighted inputs being used as inputs to the so-called activation function, which maps the input to the output either directly (identity activation), within certain limits (sigmoid, or tanh), or maps it while removing unwanted values (e.g., ReLU which removes negative values, and maps positive ones directly) [24]. The weights of the neuron connections are initially random, but then adjusted through the backward propagation process, in which the error for a forward propagated of the MLP results gets back-propagated through, and weights are adjusted proportionally to the error [25].

Due to the fact that MLP regressor can only regress a single value, if the problem consists of multiple output values, a modular model consisting of multiple models must be used. While similarities are possible between models; training the models completely separately means that all the architectures will be tested, giving a higher chance to finding a better prediction model for each goal. In the research presented, three separate MLPs are trained—one for each of the goals—infected, recovered, and deceased patients.

To confirm the validity of the results, the cross-validation process has been performed. The cross-validation method used in this research is the K-Fold algorithm [22, 26]. During this process, the dataset is split into *k* subsets (in presented case *k* = 5). Then, each of them is used as a testing set, while the remaining *k* − 1 subsets are used as a training dataset [27]. The result is then presented as the average of achieved scores, with standard deviation noted.

The solution has been implemented using Python 3.8 programming language, using scikit-learn library [28]. Scikit-learn has been selected due to ease of use, as well as the fact that it contains the implementation of most of the methods used in this research [29]. ActiveState ActivePython implementation of Python and needed libraries has been used [30]. Training has been performed using a high-performance computer (HPC)—Bura Supercomputer. To train the models 16 HPC nodes, each containing 48 logical CPUs (24 physical cores on Intel Xeon E5), with 64 GB of RAM per each node [31]—resulting in total of 768 logical CPUs used. The operating system used is Red Hat Enterprise Linux, with kernel version 3.10.0-957.

### 2.3. Hyperparameter Determination

Hyperparameters are values which define the architecture of the ANN model. Correct values of hyperparameters are crucial in achieving a quality model. To determine the best hyperparameter combination, the grid search algorithm has been used.

The grid search algorithm takes a set of possible parameters for each of the adjusted hyperparameters. Then, each possible combination of hyperparameters is determined [32]. Each of the combinations is used to train the MLP. To avoid the possibility of poor solutions due to the initial random setting of the weights, each set of hyperparameters is used for training three times. Each of the achieved models is then evaluated. The hyperparameters adjusted in performed research are [28, 29]:

- solver—the algorithm used for recalculating the weights of the MLP during back propagation process in training
- initial learning rate *α*—value of learning rate at the beginning of training
- adjustment of learning rate—the way the learning rate will change during the training, and if it will be adjusted depending on the current value of cost function or not
- number of hidden layers and neurons—defined as tuple, in which each integer defines a single hidden layer and the integer value defines the number of neurons in that layer
- activation function—function used to transform the input values of the neuron to the output value of the neuron, and
- regularization parameter L2—parameter which limits the influence of input parameters, to avoid the ANN being trained with a bias towards a single input value which has a high correlation to the output; larger the parameter, more is the influence lowered

Possible hyperparameter values are given in Table 1.

### 2.4. Model Quality Estimation

Every obtained model is evaluated using the coefficient of determination (*R*2). The coefficient of determination defines how well is the variance which exists in the real data explained with the predicted data. The real output data, the actual number of patients, is contained in the vector *y*, while the predicted data, obtained from the trained model, is set into the vector ˆ*y*. With that, the coefficient of determination *R*2 can be determined as the coefficient between the residual variance and total variance [33]:

(1) $$\begin{array}{c} R^{2}=1-\frac{S_{RESIDUAL}}{s_{TOTAL}}=1-\frac{\sum_{i=0}^{m}{\left(y_{i}-{\hat{y}}_{i}\right)}^{2}}{\sum_{i=0}^{m}{\left(y_{i}-1/m\sum_{i=0}^{m}y_{i}\right)}^{2}}, \end{array}$$

with *m* being the number of evaluated samples (length of vectors *y* and ˆ*y*). *R*2 is defined in the range *R*2∈ [0,1], with the value of 0.0 meaning that none of the variances in real data is explained in the predicted data, and the value of 1.0 being the best possible value, meaning all of the variances is explained in the predicted data.

Due to cross-validation being used, each architecture is trained 5 times—on differing data. To present the results of cross-validation, the average of *R*2 scores is calculated $\left({\bar{R}}^{2}=1/5\sum_{i=1}^{5}R_{i}^{2}\right)$. To show the variance between the scores on different folds, the standard deviation of the *R*2 scores is also presented $\left(\sigma =\sqrt{\sum_{t=1}^{5}\left(R_{t}^{2}-{\bar{R}}^{2}\right)/5}\right)$.

## 3. Results and Discussion

In this section, the detailed descriptions of the achieved results are presented. These results were achieved using the methodology described in the previous section. After the presentation of the results, the results are discussed.

### 3.1. Results

Best models achieved show a high-quality regression, with *R*2 scores of 0.98599 for the confirmed patient model, 0.97941 for the recovered patient model, and 0.99429 for the deceased patient model.

The best models achieved for all three goals (number of infections, recoveries, and deaths) have a same basic ANN architecture. These architectures consist of four hidden layers, and 16 total hidden neurons distributed equally among layers—4 neurons each.

Best models for all three outputs also use the ReLU activation function and the LBFGS solver. The best model for confirmed cases has a constant learning rate of 0.1 and has a regularization parameter of 0.0001. For the recovered cases, MLP uses a constant learning rate of 0.5 and a regularization parameter of 0.001. The model for predicting the number of deceased patients uses the adaptive learning rate of 0.01, with the regularization parameter set at 0.1. The hyperparameters of the best models are listed in Table 2.

**Figure 3:**
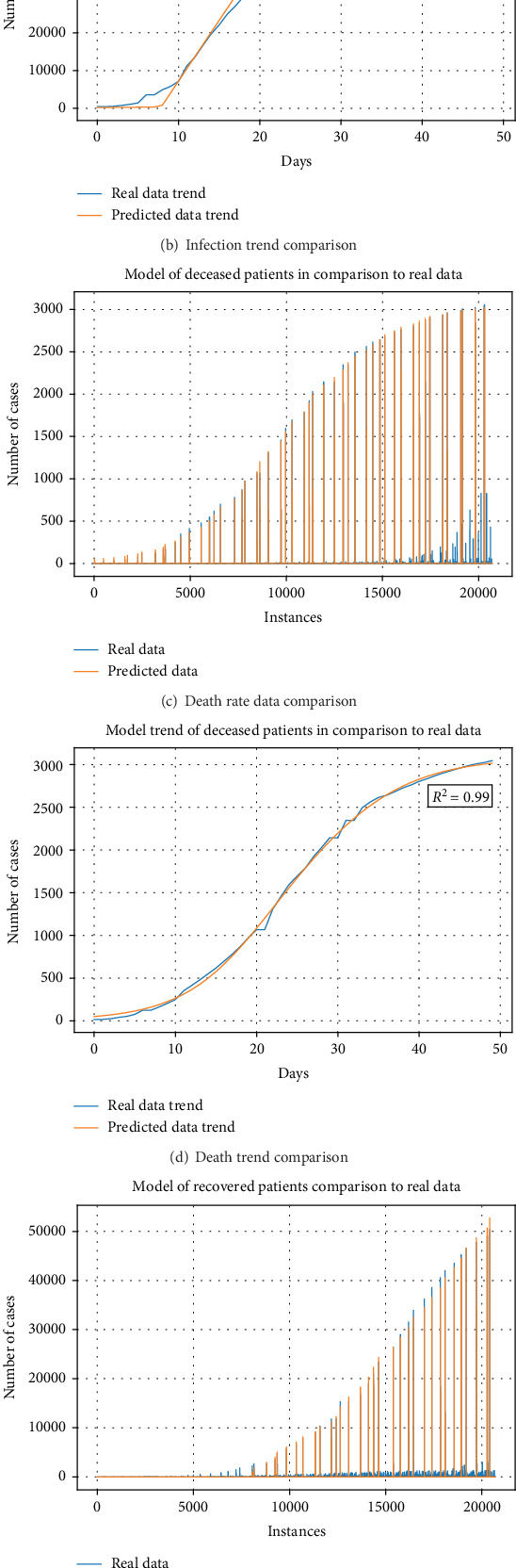
Comparison of real and modeled data. Comparison of the number of cases for each input into the dataset are shown for infected (a), deceased (c), and recovered (e) patients, while the trend of the data and model through the days analyzed are shown for the infected (b), deceased (d), and recovered (f) patients.

Figure 3 shows the comparison of real data to data obtained from the model. Real data, sorted by days, as well as trends for all three modeled cases, are shown in subfigures. Subfigures (a), (c), and (e) demonstrate the comparison of real data, sorted by date for various locations and the data predicted by model. Each bar presents a number of patients in a given group, per location. For easier viewing, the maximum of each daily count is plotted as the envelope of the plotted data in (b), (d), and (f). These envelopes show an approximation of maximal disease spread per patient group, for both real data and modeled data, which shows that the modeled data follows the collected data closely.  Table 3 shows the cross-validation results achieved for the best models shown in Table 2.

Training time, using 5-fold cross-validation, on the system used and described in the “Materials and Methods” section is shown in Table 4. Taking into account 5376 training items, and training repeated 5 times due to cross-validation, for a total of 26880 models trained, this means that the average model training time is 0.088 minutes or 5.26 seconds.

### 3.2. Discussion

Results show that a similar architecture can be used for all three models, suggesting a similar trend between all three goals. The use of the ReLU activation function is not unexpected, as it eliminates the negative values, it is logical it is going to lend itself well to a model which predicts only positive values. Learning rates differ between models, both the models for infected and recovered use a relatively high constant learning rate, while the deceased model uses a significantly lower learning rate but adapts over iterations. The regularization parameter is relatively low for the model of infections but raises for the recovered and deceased models—pointing to the fact that there is a higher influence of certain input parameters on the output of those models which needed to be suppressed.

Models show poor tracking of sudden and unexpected changes, such as the sudden jump in infections around day 22. Still, the model demonstrates good tracking of overall model change, giving good predictions even after such unexpected leaps—if given time to adjust. Due to the largest number of cases being located in China, the model is largely fitted to that data. Future changes in the maximum number of infected, deceased, or recovered patients should be included in the model to further test its robustness.

Cross-validation performed shown across the solution space shows a drop in *R*2 scores. The model for deceased patients shows the lowest drop in scoring used. The model of confirmed cases shows a more significant drop from 0.986 to 0.94, but these results are still acceptable. The highest drop is shown in the model of recovered patients where *R*2 score drops from 0.97941 to 0.781, showing the low robustness of the model for recovered patients. The architectures of the models that show the best results remain the same when cross-validation is applied.

The aim of this research, which was to generate a model of coronavirus disease spread on a global level using machine learning methods, was achieved. The created models show a high fidelity to existing data, with the exception of the model for recovered patients. In comparison to already designed models, presented models show a higher accuracy, as well as tracking of deaths and recoveries. Additionally, the presented model is created using a simpler AI algorithm and uses a comparatively simple architecture, which has performance benefits in terms of computational time and resources [22]. Results demonstrate a clear ability to mathematically model a spread of an infective disease using AI on a relatively limited dataset, meaning that comparatively long periods of data collection are not strictly necessary to achieve a good model with AI algorithms. Obtained results point towards the ability to use such algorithms to model similar phenomena in the future.

## 4. Conclusion

The achieved models show that it is possible to acquire a quality model of novel viral infections using AI methods, with geographical and time data as inputs. In this research, high accuracy models have been achieved for all regression goals. Achieved results prove the fact that AI models can be used in modeling problems such as the spread and effect of infectious diseases. This means that the application of AI methods should be attempted in modeling the present and future spread of infective diseases, in an attempt to predict the impact of such infections on humankind. Model fitting to largely the Chinese patient population shows that using the number of patients per country is not necessarily a good metric to use as a training goal—further research should be invested in testing how different types of metrics (e.g., percentage of disease in population) affect model quality. The code and models achieved can be found at a public repository, made available by the authors [34]. Authors are also planning on the implementation of achieved models inside an easy to use and widely accessible web-interface.

Future work should apply other methods in an attempt to find even better models or models that are simpler to use, or more transparent than ones observed with MLP. Comparison of models for different infective diseases would be interesting. More data being acquired should enable the use of other techniques such as recurrent neural networks to be applied on the analyses of infection models using time-series data.

## Figures and Tables

**Table 1:** Hyperparameters used in training. First column lists the hyperparameter name, while the possible values of the hyperparameter are listed in the second column. The last column presents the number of hyperparameters, with the last row showing the total number of hyperparameter combinations, obtained and used during the grid search algorithm execution.

Hyperparameter	Possible values	Count
Solver	Adam, LBFGS	2
Initial learning rate	0.00001, 0.01, 0.1, 0.5	4
Learning rate adjustment	Constant, adaptive, invscaling	3
Hidden layer sizes	(3), (6), (4, 4), (3, 3, 3), (6, 6, 6), (4, 3, 4), (12, 12, 12), (4, 4, 3, 3), (4, 4, 4, 4), (6, 6, 6, 6), (10, 5, 5, 10), (3, 3, 3, 3, 3), (10, 10, 10, 10, 10), (12, 12, 6, 6, 3, 3)	14
Activation functions	ReLU, identity, logistic, tanh	4
Regularization parameter	0.00001, 0.001, 0.01, 0.1	4
Total number of hyperparameter combinations		5376

**Table 2:** Hyperparameters of MLPs for best models achieved. Each column is one of the models—predicting the number of infected, recovered, and deceased patients. Hyperparameters that resulted in the best model are shown in rows.

Hyperparameter	Infected model	Recovered model	Deceased model
Solver	LBFGS	LBFGS	LBFGS
Initial learning rate	0.1	0.5	0.01
Learning rate adjustment	Constant	Constant	Adaptive
Hidden layer tuple	(4, 4, 4, 4)	(4, 4, 4, 4)	(4, 4, 4, 4)
Activation function	ReLU	ReLU	ReLU
L2 regularization parameter	0.0001	0.001	0.01

**Table 3:** The results of k-fold cross-validation, (*k* = 5). Average scores for each goal and the standard deviation are shown.

Goal	Average *R*^2^ score across folds	*σ*
Confirmed	0.94	0.037
Recovered	0.781	0.072
Deceased	0.986	0.021

**Table 4:** Training times in minutes for each goal, using 5-fold cross-validation and grid search of 5376 items. Training time measured using 16 48-thread HPC nodes. Average training time across all goals is shown in the bottom.

Goal	Training time (min)
Confirmed	2428
Recovered	2436
Deceased	2209
Average	2357.67

## Data Availability

This research uses a publicly available dataset “2019 Novel Coronavirus Data Repository” published by Johns Hopkins University Center for Systems Science and Engineering (JHU CSSE) available at: https://github.com/CSSEGISandData/COVID-19. Models achieved, and the code used in their generation are available in a repository, located at: https://github.com/RitehAIandRobot/COVID-19-MLP [34].
